# Differences in Femoral Artery Occlusion Pressure between Sexes and Dominant and Non-Dominant Legs

**DOI:** 10.3390/medicina57090863

**Published:** 2021-08-24

**Authors:** Nicole D. Tafuna’i, Iain Hunter, Aaron W. Johnson, Gilbert W. Fellingham, Pat R. Vehrs

**Affiliations:** 1 Department of Exercise Sciences, Brigham Young University, Provo, UT 84602, USA; nicoletafunai@gmail.com (N.D.T.); iain_hunter@byu.edu (I.H.); wayne_johnson@byu.edu (A.W.J.); 2 Department of Statistics, Brigham Young University, Provo, UT 84602, USA; gilbert_fellingham@byu.edu

**Keywords:** resistance exercise, blood flow restriction, blood flow restriction exercise

## Abstract

*Background and Objectives*: Blood flow restriction during low-load exercise stimulates similar muscle adaptations to those normally observed with higher loads. Differences in the arterial occlusion pressure (AOP) between limbs and between sexes are unclear. We compared the AOP of the superficial femoral artery in the dominant and non-dominant legs, and the relationship between blood flow and occlusion pressure in 35 (16 males, 19 females) young adults. *Materials and Methods*: Using ultrasound, we measured the AOP of the superficial femoral artery in both legs. Blood flow at occlusion pressures ranging from 0% to 100% of the AOP was measured in the dominant leg. *Results*: There was a significant difference in the AOP between males and females in the dominant (230 ± 41 vs. 191 ± 27 mmHg; *p* = 0.002) and non-dominant (209 ± 37 vs. 178 ± 21 mmHg; *p* = 0.004) legs, and between the dominant and non-dominant legs in males (230 ± 41 vs. 209 ± 37 mmHg; *p* = 0.009) but not females (191 ± 27 vs. 178 ± 21 mmHg; *p* = 0.053), respectively. Leg circumference was the most influential independent predictor of the AOP. There was a linear relationship between blood flow (expressed as a percentage of unoccluded blood flow) and occlusion pressure (expressed as a percentage of AOP). *Conclusions*: Arterial occlusion pressure is not always greater in the dominant leg or the larger leg. Practitioners should measure AOP in both limbs to determine if occlusion pressures used during exercise should be limb specific. Occlusion pressures used during blood flow restriction exercise should be chosen carefully.

## 1. Introduction

Blood flow restriction (BFR) applied to the arms or legs during low-load resistance training is effective in promoting hypertrophy and increasing or maintaining muscle strength [1,2,3,4,5]. Blood flow restriction exercise (BFRE) can be part of musculoskeletal rehabilitation following an injury or surgery or for those trying to counter muscle wasting due to chronic disease [4,6,7]. The muscular adaptations to BFRE contribute to the popularity of this method of resistance training among athletes and in the fitness industry.

Blood flow restriction partially restricts arterial blood flow into the limb and occludes venous blood flow out of the muscle [6,7,8,9]. Some studies [10,11] used elastic wraps as a “practical” method of BFR, but this could produce inconsistent blood flow restriction between two limbs and between exercise sessions. Most studies have restricted blood flow with an inflatable cuff. Early studies used absolute cuff pressures [7,8,12,13,14,15,16] ranging from 50 to 300 mmHg, but this is problematic in that a given cuff pressure represents a different level of occlusion and blood flow restriction for each person. The current recommendation [5,17] is to use a percentage of the arterial occlusion pressure (AOP) to restrict blood flow during BFRE. Although further research is needed to determine the optimal pressure to use during BFRE, it appears that a pressure equivalent to 50% to 80% of the AOP is appropriate during low-load resistance training [5].

Although a plethora of papers have been published on the topic of BFR and BFRE, some things remain unclear, including the relationship between blood flow and occlusion pressure, sex differences in AOP, and differences in AOP between dominant and non-dominant limbs. Differences in the AOP between individuals is attributed primarily to differences in limb circumference [5,18]. To date, studies have not reported the AOP in the dominant and non-dominant limbs in an individual. In most of the literature where occlusion pressure is based on a percentage of AOP, dominance of the occluded limb is not reported in unilateral interventions and any differences between limbs are not reported in bilateral studies [19,20,21,22,23,24]. Any differences in AOP between the dominant and non-dominant limbs may be due to differences in limb circumference. Although previous studies have included male and female participants, sex differences in AOP have not been reported. After accounting for potential differences in limb circumference, little evidence suggests that there is a sex difference in AOP. Some authors have reported that the relationship between arterial blood flow and absolute cuff pressure in the leg is linear [7,8,12,25] but a recent study [26] reported a nonlinear relationship between relative arterial blood flow and cuff pressures with a plateau in blood flow at pressures between 40–80% AOP. 

The purposes of this study were to compare the AOP of the superficial femoral artery (SFA) in the dominant and non-dominant legs, and blood flow at relative occlusion pressures (0–100% AOP) in the dominant leg at rest in young healthy men and women. We hypothesized a direct positive relationship between limb circumference and AOP, no significant difference in AOP between the sexes or between the dominant and non-dominant legs, and no significant sex difference in the relationship between arterial blood flow and relative occlusion pressure.

## 2. Materials and Methods

This study was a cross-sectional study that measured the AOP in the SFA of both legs and blood flow in the dominant leg at cuff pressures representing 0%, 20%, 40%, 60%, 80%, and 100% of the AOP. The primary variables of interest included the AOP (mmHg) and blood flow at each increment of blood flow restriction (% AOP). This study was reviewed and approved by the Institutional Review Board for the use of Human Subjects prior to the collection of any data.

### 2.1. Participants

A total of 35 (16 males, 19 females) physically active and apparently healthy adults, 18–35 years of age participated in this study. Interested participants were excluded from participation if they had any known risk factors for cardiovascular disease or one or more risk factors for thromboembolism, which include: obesity (BMI ≥ 30 kg/m^2^), diagnosed Crohn’s disease, a previous fracture of the hip, pelvis, or femur, a major surgery in the last 6 months, varicose veins, a family history of deep vein thrombosis or pulmonary embolism, and on oral birth control [22,27,28,29]. Individuals were also excluded if a) they had been diagnosed as having or were being treated for cardiovascular disease, renal disease, diabetes, or hypertension, b) their resting systolic blood pressure (SBP) ≥ 130 or diastolic blood pressure (DBP) ≥ 80 mmHg, or c) they were pregnant or less than 6 months postpartum. To minimize the effects of hormone variability later in the menstrual cycle, females participated in the study within the first 14 days of their menstrual cycle.

### 2.2. Procedures

Subjects were instructed to refrain from eating during the 2 h prior to their participation, consuming caffeine for the previous 8 h, and participating in vigorous physical activity the previous 24 h [28,29]. All procedures for each subject were completed in one visit to the lab. The methods, expectations, risks, and benefits of the study were explained to each subject after which they voluntarily provided written informed consent. 

The subject’s height (cm) was measured using a calibrated wall-mounted stadiometer scale (SECA Model 264; SECA, Cino, CA, USA). Body mass (kg) was measured using a digital scale (Ohaus Model CD-33, Ohaus Corporation, Pine Brook, NJ, USA) and BMI (kg/m^2^) was calculated from measured height and body mass values. Subjects then sat quietly in a comfortable chair for 5 min with legs uncrossed. Blood pressure was measured on the right arm and the average of two blood pressure measurements was recorded, or if they were not within 5 mmHg of each other, blood pressure was measured a third time, and the two closest measurements were averaged. Mean arterial pressure (MAP) was calculated as DBP plus one-third of pulse pressure. Leg dominance was determined by self-report by asking “I you were to kick a ball, with which leg would you use to kick the ball? [30]. The circumference and skinfold thickness of the dominant and non-dominant thighs were measured in triplicate in the standing position using a spring-loaded Gullick measuring tape and a calibrated Lange caliper (Santa Cruz, CA, USA), respectively. Measurements were taken at one-third of the distance between the inguinal crease and the top of the patella. The average of the three measurements was used in the data analysis. 

### 2.3. Blood Flow Measurements

All blood flow measurements were performed using a handheld Doppler probe (9 MHz; 55 mm) and GE ultrasound machine with an integrated ECG (GE LOGIQ, GE Healthcare). Blood flow restriction was accomplished using a Hokanson SC10 cuff (10 cm wide; 85 cm long) attached to an E-20 rapid cuff inflator (Hokanson, Bellevue, WA, USA). The occlusion cuff was placed on the participant’s thigh one-third of the distance between the inguinal fold and the top of the patella and blood flow in the SFA was measured distal to the cuff. Color flow mode and pulse wave forms were viewed to determine the presence of blood flow. During the entire time of testing, participants were in a semi-reclined (15°) position to allow reasonable access to the SFA using the ultrasound. Angle of insonation of the ultrasound probe was maintained at 60°.

### 2.4. Measurement of Arterial Occlusion Pressure

The AOP of the SFA in the dominant and non-dominant legs was measured once in a randomized order for each participant. A hand-held Doppler probe was used to detect a pulse wave in the SFA distal to the cuff with the cuff deflated. The cuff was then inflated to 50 mmHg and then gradually increased until arterial flow and pulse waves were no longer detected. After the AOP was recorded, the cuff was deflated, removed, and placed on the other leg. The participant rested for 5 min [20,27,29] with the cuff deflated, after which the process was repeated.

### 2.5. Measurement of Arterial Blood Flow

Following at least 5 min after the second AOP measurement, we measured arterial blood flow for 1 min at cuff pressures equivalent to 0%, 20%, 40%, 60%, 80%, and 100% of the subject’s previously measured AOP in a randomized order. There was a 5 min rest period between measurements with the cuff deflated. One-minute video clips were stored for later analysis. Using the integrated ECG and pulse waves as reference points, femoral artery diameter was measured at two time periods representing the end of diastole (just before the QRS) and during systole (at the peak of the QRS) of each cardiac cycle. The two measurements were averaged for each beat over five 12-s periods. Time averaged blood flow velocity (TAV) over the five 12-s periods was recorded. Blood flow (mL/min) was calculated automatically by the ultrasound machine as follows:

Blood flow (mL/min) = Cross sectional area (cm^2^) × TAV (cm/s) × 60 s/min

### 2.6. Data Analysis

Sex differences in age, height, body mass, BMI, blood pressure measurements (i.e., SBP, DBP, MAP), leg circumference, thigh skinfold thickness, and AOP in the dominant and non-dominant legs were determined using two-sample *t*-tests. Differences in leg circumference, thigh skinfold thickness, and AOP between the dominant and non-dominant legs in males and females were determined using paired *t*-tests. The influence of sex, SBP, DBP, MAP, thigh skinfold and circumference measurements on the AOP was evaluated using regression analysis.

Analysis of arterial blood flow data, expressed as a percentage of unoccluded blood flow, and occlusion pressure, expressed as a percentage of individual AOP (0%, 20%, 40%, 60%, 80%, 100%) presented two major challenges. The first was that relative blood flow when there was no occlusion (0% AOP) is represented as 100% for every subject and there is no variance in the data. Second, blood flow at various degrees of occlusion (e.g., 20%, 40%, 60% AOP) for some subjects was higher than when there was no occlusion (0% AOP). Thus, the difficulty in analyzing the blood flow data was that there was one data point (0% AOP) where there is no variance in blood flow (blood flow = 100%) and other data points where relative blood flow was greater than that measured at 0% AOP (blood flow = 100%). To analyze these data, we first used a one-sample *t*-test to determine if relative blood flow at 20% AOP was significantly different from relative blood flow at 0% AOP. We found that the average relative blood flow at 20% AOP was 81% (CI = 70.4−91.6%) of unoccluded blood flow (*p* = 0.0009). Since each subject had multiple data points, we then fit a mixed linear model between relative blood flow and relative occlusion pressure to account for within- and between-subject variability. To further appropriately account for variability when fitting the model, we omitted the blood flow data at 0% AOP and only used data at occlusion pressures of 20%, 40%, 60%, 80%, and 100% of AOP. The initial analysis revealed that there was no sex difference in the relationship between blood flow and occlusion pressure. We therefore fit a linear model that did not include sex as a variable. A 95% confidence interval (CI) and prediction interval (PI) were computed for the line of best fit through the data.

## 3. Results

Participant characteristics are shown in Table 1. Males were taller, heavier and had higher SBP and MAP than their female counterparts. There were no significant differences in the circumferences of the dominant and non-dominant legs in males (*p* = 0.1897) or in females (*p* = 0.0895) or of the dominant (*p* = 0.847) or non-dominant legs (*p* = 0.746) between males and females. There were no significant differences in the thigh skinfold between the dominant and non-dominant legs of either males (*p* = 0.7630) or females (*p* = 0.5923) or of the dominant leg (*p* = 0.056) and non-dominant leg (*p* = 0.054) between males and females. 

### 3.1. Arterial Occlusion Pressure

**Figure 1:**
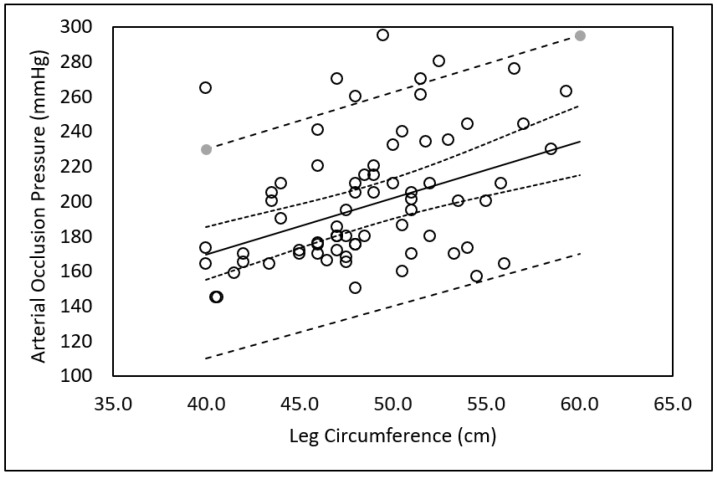
Relationship between limb circumference and arterial occlusion pressure. Solid line = line of best fit. Dashed lines = 95% Prediction Intervals. Dotted lines = 95% Confidence Intervals.

There was a significant difference in the AOP between males and females in the dominant (230 ± 41 vs. 191 ± 27 mmHg; *p* = 0.002) and non-dominant (209 ± 37 vs. 178 ± 21 mmHg; *p* = 0.004) legs, respectively. There was a significant difference in the AOP between the dominant and non-dominant legs in males (230 ± 41 vs. 209 ± 37 mmHg; *p* = 0.009) but not in females (191 ± 27 vs. 178 ± 21 mmHg; *p* = 0.053). Regression analysis revealed that after leg circumference entered the equation, SBP, DBP, MAP, skinfold thickness, age, and sex were not significant independent predictors of AOP. The resulting regression model as shown in Figure 1 with 95% CI and 95% PI was:

AOP (mmHg) = 40.4 + (3.23) Leg Circumference (cm)

### 3.2. Arterial Blood Flow

**Figure 2:**
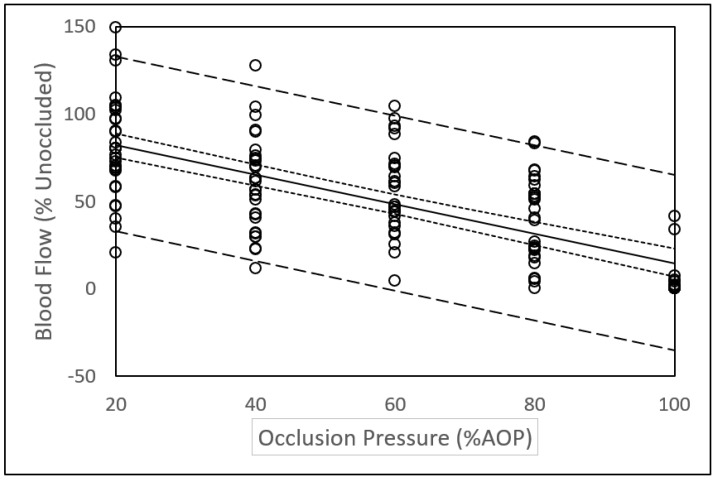
Relationship between arterial blood flow and occlusion pressure. Solid line = line of best fit. Dashed lines = 95% Prediction Intervals. Dotted lines = 95% Confidence Intervals.

The mixed model analysis revealed a linear relationship between relative blood flow (% unoccluded blood flow) and relative occlusion pressure (%AOP). The resulting equation (R = −0.842; Residual Standard Error = 25.3) as shown in Figure 2 with 95% CI and 95% PI was:

Percent Blood Flow = 99.46 − 0.85 (Occlusion Pressure; %AOP)

## 4. Discussion

This paper adds to the current body of knowledge about BFR in that we report, perhaps for the first time, large differences in AOP between males and females and between the dominant and non-dominant legs. The linear relationship between blood flow and occlusion pressure expressed in relative terms was unrelated to sex. We also report a large variance in blood flow data at different levels of occlusion that is not unique to this study but has not been previously discussed. The findings of this study have implications for future research and those using BFRE.

### 4.1. Sex and Limb Differences in Arterial Occlusion Pressure

We report a large sex difference in AOP in both the dominant and non-dominant legs (Table 1). Although other studies have included male and female participants, sex differences in AOP have not been reported [21,26,31,32]. To the best of our knowledge, only one previous study has reported a sex difference in AOP. Jessee et al. [27] reported that the AOP of the right arm of females was on the average 4–7 mmHg (*p* < 0.05) lower than in males across three different cuff sizes. Although significantly different, the authors suggest that the differences in AOP were inconsequential in prescribing BFRE. 

It is well reported that differences in the AOP can be attributed primarily to differences in limb circumference [7,18,33]. The larger the limb, the greater the pressure required to occlude the blood vessel. Larger limbs have more mass between the skin and the blood vessels that must be compressed to occlude the vessel, and higher pressures are required to transmit adequate force to the deeper tissues [20,21]. Hence, it follows that sex differences in AOP or differences in AOP between limbs may be accounted for by differences in limb circumference. Jessee et al. [27] reported an average sex difference in circumference of the right arm of 5.3 cm and that after accounting for arm circumference, arm length, SBP, and DBP, sex remained a significant independent predictor of AOP. In this study, there was an average difference in circumference of <1 cm in both the dominant and non-dominant legs between and within males and females (Table 1). Despite a small average difference in leg circumference, there was a large difference in AOP between the dominant and non-dominant legs within and between males and females (Table 1). This could be attributed to the fact that the difference in the circumferences of the dominant and non-dominant leg ranged from the dominant leg being 5.5 cm smaller to 7 cm larger than the non-dominant leg in males and 2.5 cm smaller to 3.5 cm larger in females. The regression analysis in this study indicates that after accounting for leg circumference, SBP, DBP, MAP, skinfold thickness, sex, and age were not significant predictors of AOP. The differences in the results between this study and that of Jessee et al. [27] might be explained by the differences in the size of the limbs studied (i.e., legs vs. arms). In addition, we report the AOP of both the dominant and non-dominant legs, whereas Jessee et al. only reported the AOP of the right arm, rather than the dominant arm. It should be appreciated that the leg circumference of the dominant leg is not always larger than that of the non-dominant leg. In this study, the dominant leg was larger than the non-dominant leg in 20 of the participants (8 males, 12 females) and the non-dominant leg was larger in 15 of the participants (8 males, 7 females). Likewise, the AOP is not always higher in the larger leg. In this study, the AOP was higher in the larger leg of 21 participants (15 dominant, 6 non-dominant; 9 males, 12 females) and higher in the smaller leg of 14 participants (9 dominant, 5 non-dominant; 7 males, 7 females).

Considering that the overall average difference in AOP between the dominant and non-dominant legs in this study was 17 mmHg (Table 1), an occlusion pressure of 50% of AOP would result in a difference in occlusion pressure of less than 9 mmHg between legs. This small difference in occlusion pressure between the two limbs would be of little import during BFRE. Nevertheless, the greatest difference in AOP between the two legs in an individual in this study was 80 mmHg. In this subject, the difference in occlusion pressure between the two legs during BFRE would be of practical significance. Our data suggest that AOP should be measured in both legs to determine if a sufficient difference existed that would justify using occlusion pressures specific to each leg. Most practitioners (e.g., physical therapists, personal trainers, strength and conditioning coaches, etc.) are unable to measure AOP in their clients or patients. Nevertheless, health and fitness professionals should be aware of differences between limbs that could affect the safe use of BFR during exercise. End-users should not be naïve of the potential differences between limbs and should use BFR during exercise with appropriate caution. Although further research is needed, occlusion pressures at a perceived pressure [10,11] could account for difference between limbs when measures of occlusion pressure are not possible.

The composition of the limb may also influence the pressure required to occlude a blood vessel. In this study, the sex differences in thigh skinfold thickness approached the alpha-level of 0.05 but likely did not enter into the regression equation to estimate AOP because it is included as part of the overall circumference of the leg and represents only a portion of the total tissue mass that must be compressed to occlude the femoral artery. Our data concur with that of Loenneke et al. [21] who, after using B-mode ultrasound to measure fat thickness of the upper arm of 171 males and females, concluded that the absolute size of the arm may be more important than the composition of the arm in predicting AOP.

### 4.2. Arterial Blood Flow

Since blood flow at any given absolute pressure varies widely between individuals, it is appropriate to express blood flow and occlusion pressure in relative terms. The results of this study (Figure 2) indicate a linear relationship between blood flow (% unoccluded blood flow) and relative occlusion pressure (%AOP). Our data concur with those of a previous study reporting a linear relationship between relative blood flow and %AOP in the posterior tibial artery [25]. This is contrary to recently reported nonlinear relationships between relative blood flow and relative occlusion pressure and plateaus in blood flow between 40% to 80% AOP in the brachial artery [34,35] and the SFA [26]. Some difference in methodology between studies could help explain the disparity in the results. For example, subjects in our study were in a semi reclined position, whereas subjects in Crossley et al. [26] study were in the seated position. Additionally, measurements in the study by Crossley et al. were performed on alternating legs in a randomized order over the course of the study so the reported AOP and blood flow represented AOP and blood flow in both legs rather than either the dominant or non-dominant leg. 

Whether the relationship between blood flow and occlusion is linear or nonlinear is of practical importance. A nonlinear relationship suggests that use of a lower, more comfortable and potentially safer occlusion pressure (e.g., 40% AOP) would provide an equally effective stimulus during BFRE as higher occlusion pressures. A linear relationship suggests that the occlusion pressure used during BFRE should be selected more carefully and that further research is required to determine a recommended reduction in blood flow to be used during BFRE.

### 4.3. Variance in Blood Flow Measurements

In this study, we observed a large variation in blood flow at different levels of occlusion. For example, we note that blood flow at higher levels of occlusion was sometimes greater than at lower levels of occlusion. We also found that some participants had notable blood flow at an occlusion pressure equivalent to the previously measured AOP. Variance in the data presented in this study is apparent in the wide prediction intervals shown in Figure 2. These observations are suggestive of a robust cardiovascular system that maintains blood flow across various levels of occlusion pressures [35]. 

Evidence of the variation in blood flow measurements is present in previous studies. For example, close examination of previously reported blood flow data [34] reveals that relative blood flow at 70% AOP was greater than relative blood flow at 60% and 50% AOP. Likewise, previously reported large standard deviations of blood flow data [35] suggest that in some subjects, blood flow at higher occlusion pressures was greater than at lower occlusion pressures. This could be attributed to a cardiovascular response to high occlusion pressures in the absence of exercise [7,36]. It could also be possible that after several applications of BFR there are local responses in the vasculature that alters blood flow or the AOP. Although previous research indicates that blood flow returns to normal within 30 to 90 s after the occlusion is removed [37], longer rest periods may be needed between sequential blood flow measurements with occlusion. It is possible that after multiple occlusions of blood flow, the AOP changes. This could influence the expression of blood flow relative to AOP. Lastly, although data collected from each subject in this study occurred in a single day, Mouser et al. [35] reported a significant day-to-day variation in resting blood flow that clearly has implications for future research involving blood flow measurements over multiple days and the use of BFRE. Our data and close examination of data presented in the literature warrants a call for further studies evaluating the variance and reliability of blood flow measurements during BFR.

### 4.4. Study Limitations

This study had several limitations. Participants were college-age coeds without known risk factors for cardiometabolic diseases. Therefore, the results of our study may not be applicable to all populations. Blood pressure was not measured during the measurement of blood flow at different occlusion pressures. Having blood pressure measurements could lead to a better understanding of the relationship between occlusion pressure and blood flow. Blood pressure measurements during blood flow occlusion may also help explain the variation in blood flow at different occlusion pressures. In this study, the Hokanson SC10 cuff (10 cm wide; 85 cm long) attached to an E-20 rapid cuff inflator (Hokanson, Bellevue, WA, USA) was used for all measurements. Clinicians, researchers, and other practitioners may use different brands of cuffs and inflation systems, different cuff sizes, or other methods to occlude blood flow. Lastly, a greater number of subjects could improve the data when comparing limb and sex differences in AOP.

### 4.5. Direction for Future Studies

Based on the results of this study, future studies should include both male and female participants and report limb dominance, AOP, and blood flow data on both limbs in males and females. Limb circumference or other measures of limb volume should also be reported. While it is clear that limb circumference is more influential on AOP than limb composition, the sex differences in thigh skinfold thickness reported in this study and the influence of fat thickness reported by Loenneke et al. [21] lend support for an influence of limb composition on AOP that needs further investigation. The large variation of blood flow measurements at different occlusion pressures reported in this and previous studies suggests the need to standardize blood flow measurement methods and investigate the reliability of AOP and blood flow measurements. Comparing blood flow and variability in blood flow at different occlusion pressures between the dominant and non-dominant leg is also warranted. Measuring blood pressure during occlusion may help explain variation in blood flow at different occlusion pressures. Assessments of reliability in measurements of blood flow with and without occlusion between test administrators, within and between days needs attention.

## 5. Conclusions

An important finding of this study was large sex differences in AOP in both the dominant and non-dominant legs and large differences in AOP between the dominant and non-dominant legs particularly in men. Arterial occlusion pressure is not always greater in the dominant leg or the larger leg. Practitioners should measure AOP in both limbs to determine if occlusion pressures used during exercise should be limb specific. We also report a linear relationship between relative occlusion pressure and blood flow. The large variance in blood flow at different occlusion pressured warrants further study and caution during BFRE. These findings are of practical importance when using BFRE in various settings and suggest the need for continued research. Occlusion pressures used during blood flow restriction exercise should be chosen carefully.

## Figures and Tables

**Table 1:** Participant Characteristics.

	Males (*N* = 16)	Females (*N* = 19)	Combined (*N* = 35)
Age (years)	23.8 ± 3.6	22.9 ± 3.5	23.3 ± 3.5
Height (cm) *	177.6 ± 5.3	166.9 ± 8.7	171.8 ± 9.1
Body Mass (kg) *	75.4 ± 10.6	63.6 ± 9.6	68.8 ± 11.6
BMI (kg/m^2^)	23.9 ± 3.6	22.7 ± 2.9	23.3 ± 3.2
SBP (mmHg) *	122 ± 5.5	114 ± 5	117 ± 6.5
DBP (mmHg)	73 ± 6.4	71 ± 6	72 ± 6.3
MAP (mmHg) *	90 ± 5.7	85 ± 5	87 ± 5.6
Thigh Skinfold (mm)			
Dominant Leg	25.8 ± 11.0	32.6 ± 9.2	29.5 ± 10.5
Non-dominant Leg	25.9 ± 11.6	32.8 ± 8.8	29.6 ± 10.6
Difference	0.12 ± 1.63	0.26 ± 2.1	0.2 ± 1.87
Thigh Circumference (cm)			
Dominant Leg	48.8 ± 4.2	49.2 ± 4.9	49.0 ± 4.5
Non-dominant Leg	47.9 ± 4.5	48.5 ± 4.9	48.3 ± 4.7
Difference	0.91 ± 2.6	0.68 ± 1.6	0.78 ± 2.12
Arterial Occlusion Pressure (AOP)			
Dominant Leg *	230 ± 41	191 ± 27	209 ± 39
Non-dominant Leg *	209 ± 37	178 ± 21	192 ± 33
Difference	21 ± 28.7 ^	13 ± 27.3	17 ± 27.8

Values are mean ± SD. BMI = body mass index, SBP = systolic blood pressure, DBP = diastolic blood pressure, MAP = mean arterial pressure. * = significant difference between males and females (*p* < 0.05); ^ = significant difference between dominant and non-dominant leg (*p* < 0.05).

## Data Availability

The data presented in this study are available upon request from the corresponding author. The data are not publicly available.
